# Tissue Dissociation Workflows Influence Reactive Astrocyte Recovery in Single-Cell Transcriptomics

**DOI:** 10.1007/s12035-026-06278-w

**Published:** 2026-10-16

**Authors:** Dominika Dostalova, Pavel Abaffy, Zuzana Matusova, Jan Kriska, Tomas Knotek, Denisa Kirdajova, Jana Tureckova, Martin Pavlicek, Pavla Jendelova, Miroslava Anderova, Lukas Valihrach

**Affiliations:** 1 Laboratory of Glial Biology and Omics Technologies, Institute of Biotechnology of the Czech Academy of Sciences, Vestec, Czech Republic; 2 Faculty of Science, Charles University, Prague, Czech Republic; 3 GeneCore Facility, Institute of Biotechnology of the Czech Academy of Sciences, Vestec, Czech Republic; 4 Department of Cellular Neurophysiology, Institute of Experimental Medicine of the Czech Academy of Sciences, Prague, Czech Republic; 5 Second Faculty of Medicine, Charles University, Prague, Czech Republic; 6 Department of Neuroregeneration, Institute of Experimental Medicine of the Czech Academy of Sciences, Prague, Czech Republic

**Keywords:** Single-cell RNA sequencing, Tissue dissociation, Reactive astrocytes, Methodological artifact, Middle cerebral artery occlusion, Tauopathy, Transcriptomics

## Abstract

Single-cell RNA sequencing has advanced our understanding of glial diversity, yet tissue dissociation remains an important technical variable. We revealed that a widely used commercial dissociation protocol can underrepresent reactive astrocytes in an acute ischemic stroke model, with preliminary observations indicating a comparable trend in a chronic tauopathy model. Benchmarking against an optimized, low-shear workflow demonstrates that different tissue dissociation workflows can substantially alter the recovery and apparent transcriptomic profile of disease-associated astrocytes, selectively losing signatures of morphological remodeling. Our findings indicate that workflow-dependent recovery biases affecting reactive astrocytes may be more prevalent than recognized, underscoring the need for pathology-tailored, gentle isolation workflows to avoid distortions in downstream neuropathological interpretations and the prioritization of therapeutic targets.

**Supplementary Information:**

The online version contains supplementary material available at https://doi.org/10.1007/s12035-026-06278-w.

## Background

Astrocytes are indispensable regulators of central nervous system (CNS) health and emerge as central drivers of disease progression during reactive astrogliosis [1]. This reactive state initiates profound morphological and transcriptional transformations—most notably the upregulation of glial fibrillary acidic protein (GFAP)—across diverse acute and chronic neuropathologies [2, 3].

Single-cell RNA sequencing (scRNA-seq) has revolutionized our ability to map this astrocytic heterogeneity. Yet, a fundamental and largely unaddressed bottleneck threatens the integrity of these datasets: the physical reality of tissue dissociation. While the field has acknowledged transcriptomic artifacts stemming from sample processing delays [4] or enzymatic digestion temperatures [5, 6], a far more critical biological question remains unanswered. It is currently unknown whether specific tissue dissociation workflows differentially affect the recovery of morphologically complex reactive astrocyte populations. If critically vulnerable, disease-associated glial populations are systematically lost during single-cell isolation, our current understanding of neuropathology may be fundamentally skewed.

To resolve this critical blind spot, we benchmarked a widely used commercial dissociation protocol against two optimized in-house workflows. By evaluating both in an acute stroke model (middle cerebral artery occlusion; MCAO) alongside exploratory analysis of a chronic tauopathy model (P301S Tau; Tau), we demonstrate how standard isolation techniques severely skew astrocyte recovery and alter the transcriptional profiles of captured disease-associated populations.

## Results and Discussion

**Fig. 1:**
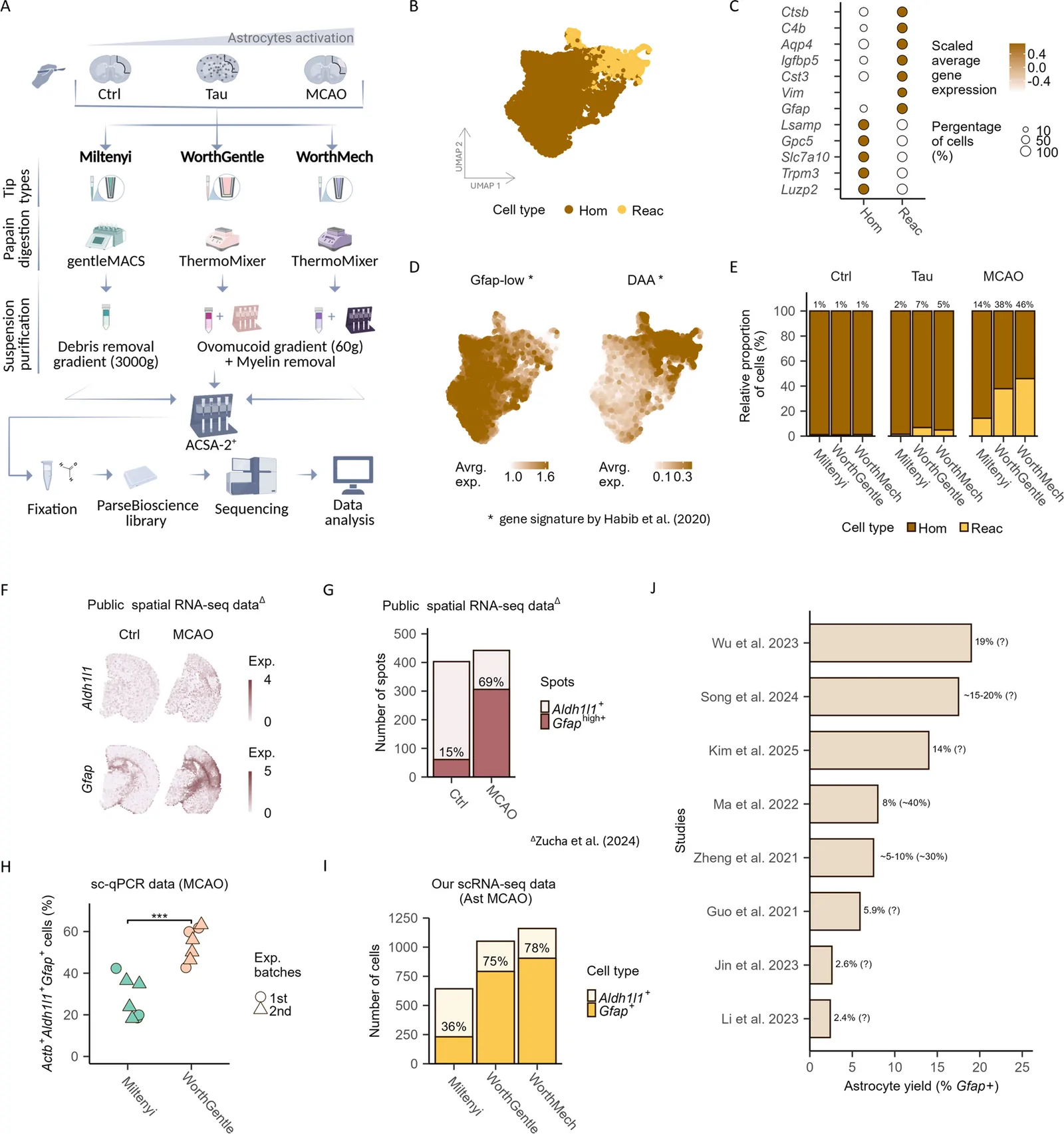
Reactive astrocytes are selectively underrepresented in scRNA-seq datasets. **A** Schematic overview of the experimental setup (created with BioRender.com). **B** UMAP projection of identified astrocyte clusters (*N* = 29 mice total, *n* = 8586 cells). **C** Dot plot of marker genes defining populations within the astrocyte cluster (*N* = 29 mice total, *n* = 8586 cells). **D** Feature plot visualizing the average expression of astrocyte gene signatures, as defined by Habib et al. [7] (*N* = 29 mice, *n* = 8,586 cells). **E** Stacked bar plots displaying the proportional abundance of astrocyte populations across experimental conditions (*N* = 29 mice total, *n* = 3 mice pooled per protocol for Ctrl and Tau, with the exception of the Tau WorthMech cohort, which comprised *n* = 2 mice; *n* = 4 mice pooled per protocol for MCAO; number of cells: Ctrl: Miltenyi *n* = 326, WorthGentle *n* = 778, WorthMech *n* = 982; Tau: Miltenyi *n* = 395, WorthGentle *n* = 1534, WorthMech *n* = 1717; MCAO: Miltenyi *n* = 643, WorthGentle *n* = 1051, WorthMech *n* = 1160). The percentage above each bar indicates the proportion of Reac astrocytes (rounded to the nearest whole integer). **F** Spatial transcriptomic visualization of *Aldh1l1* and *Gfap* expression in Ctrl and 7-day post-MCAO brain tissue (*N* = 2 mice total; Ctrl: *n* = 1 mouse, MCAO: *n* = 1 mouse). **G** Stacked bars representing absolute spot counts (*N* = 2 mice total; Ctrl: *n* = 1 mouse, MCAO: *n* = 1 mouse; number of spots: Ctrl: *n* = 403, MCAO: *n* = 442) across cortical and lesioned brain regions; internal percentages reflect the proportion of *Gfap*^high+^ spots within the *Aldh1l1*⁺ population in Ctrl and 7-day post-MCAO. **H** Comparison of isolation efficiencies for the targeted *Actb*^+^*Aldh1l1*^+^*Gfap*^+^ cell population across different workflows via sc-qPCR. Each symbol represents an individual animal (*N* = 14 mice total; *n* = 7 mice per condition) processed across two independent experimental batches (circles: 1 st batch, *n* = 3 mice per condition; triangles: 2nd batch, *n* = 4 mice per condition; 96 sort-tested single-cell reaction wells per mouse; horizontal bars show mean ± SEM). WorthGentle yielded a significantly higher recovery of reactive astrocytes (mean difference = 26.53%, 95% CI: [16.07%, 37.00%]; Welch’s *t*(11.52) = 5.55, *p* = 1.46 × 10^–4^, Cohen’s *d* = 2.97; ***p < 0.001). **I** Stacked bars showing the absolute number of *Aldh1l1*⁺ cells within the scRNA-seq astrocyte cluster from MCAO mice (*N* = 12 mice total, *n* = 4 mice pooled per protocol; number of cells: Miltenyi *n* = 643, WorthGentle *n* = 1051, WorthMech *n* = 1160); internal percentages reflect the enrichment of *Gfap*^+^ cells within *Aldh1l1*⁺ cells in Ctrl and 7-day post-MCAO. **J** Bar plot illustrating total astrocyte yields across publicly available stroke datasets. *Abbreviations*: *Ast* astrocytes, *Ctrl* control, *DAA* disease-associated astrocytes, *Gfap*^*+*^ cells with Gfap expression higher than 0, *Gfap*^*high+*^ spots with Gfap expression higher than the average Gfap expression in Ctrl, *Hom* homeostatic, *MCAO* middle cerebral artery occlusion, *Miltenyi* commercial protocol Miltenyi Biotec, *sc-qPCR* single-cell quantitative polymerase chain reaction, *Reac* reactive, *Tau* P301S tauopathy, *WorthGentle* optimized in-house protocol with minimal mechanical stress, *WorthMech* optimized in-house protocol with increased mechanical stress, *?* data not reported

To investigate how tissue dissociation impacts the capture of diverse astrocytic states, we performed scRNA-seq on ACSA-2^+^ enriched cells from MCAO and Tau models across three distinct isolation workflows: a standardized commercial protocol (Miltenyi), our optimized low-shear protocol (WorthGentle), and a modified in-house protocol with increased pipetting shear stress (WorthMech) (Fig. 1A, SFig.1A–C, STab.1). Following in silico purification (*Methods*), the captured astrocytes robustly segregated into homeostatic and reactive (Reac) clusters (Fig. 1B, C, STab. 2). These clusters closely mirrored established Gfap*-*low and disease-associated astrocyte signatures [7], confirming that all workflows successfully captured fundamentally relevant astrocytic states (Fig. 1D).

To evaluate whether isolation workflows influenced library quality or cell recovery, we assessed sample-level technical metrics across all conditions and protocols. The global quality control metrics remained consistent across all methods (SFig. 2A–C), overall cell yields ranged 915 to 4,352 quality-filtered cells per condition. In silico doublet filtering identified and eliminated between 2.1% and 10.0% high-confidence doublets per condition, while astrocyte purity ranged from 32.0% to 65.4% (SFig. 2D). Moreover, the relative abundance of these sub-clusters diverged dramatically. The Miltenyi protocol resulted in a striking underrepresentation of Reac astrocytes in the acute MCAO model compared to our in-house methods (Fig. 1E, SFig. 2E). A consistent trend was observed in the chronic Tau model (2% in Miltenyi vs. 7% in WorthGentle; Fig. 1E), although this represents a preliminary observation given the limited cohort size and low absolute Reac cell numbers. Crucially, modifying pipette-tip bore geometry to introduce standard pipetting shear within our in-house workflow (WorthMech) did not reproduce the pronounced underrepresentation observed with the Miltenyi protocol (Fig. 1E, SFig. 2E). Because these workflows represent multi-step composite pipelines differing across enzymes, mechanical disruption tools (automated gentleMACS vs. manual pestle), centrifugation speeds (3000 × *g* debris removal vs. 60 × *g* ovomucoid gradient), and buffering agents, our findings reflect cumulative differences between the end-to-end workflows rather than isolating a single physical parameter. While high-speed compaction and subsequent pellet resuspension represent plausible candidates for membrane disruption in fragile cells [8], systematic one-variable-at-a-time step-swapping experiments will be needed to definitively determine the relative contribution of centrifugation force, enzymatic sensitivity, and rotor–stator mechanical stress. Disparate enzymatic cocktails could differentially affect surface ACSA-2 (ATP1B2) preservation or capture efficiency during reactive remodeling, despite reported epitope stability [9, 10], representing another potential contributor to the observed recovery biases.

To evaluate whether this underrepresentation reflects in situ tissue composition rather than biological absence, we examined orthogonal, dissociation-free Visium spatial transcriptomics data from our matched MCAO model (day 7 post-injury) [11]. By mapping *Aldh1l1* (pan-astrocytic) and *Gfap* (reactive) expression, we observed an expansion of *Gfap*^high+^ spots—from 15% in control cortices to 69% within the MCAO lesion (Fig. 1F, G). While Visium spots represent multicellular measurements rather than single-cell resolution, this robust in situ accumulation supports the interpretation that the low proportion of Reac astrocytes recovered in the Miltenyi scRNA-seq dataset reflects a workflow-dependent recovery bias rather than a lack of Reac astrocytes in the tissue. We independently corroborated this protocol-driven bias using targeted single-cell quantitative PCR (sc-qPCR), which demonstrated a significantly higher recovery efficiency of *Actb*^+^*Aldh1l1*^+^*Gfap*^+^ cells using the WorthGentle workflow compared to Miltenyi (Fig. 1H). These targeted single-cell findings parallel the proportional distributions seen in our scRNA-seq dataset (Fig. 1I), indicating that this dissociation bias is preserved across orthogonal profiling methods. Furthermore, this sc-qPCR validation using standard reaction wells rules out the possibility that the cell loss was an artifact of the split-pool combinatorial barcoding platform used in our scRNA-seq library preparation [12]. We next examined whether the variable recovery of reactive astrocytes might be reflected across the published single-cell literature. We conducted a literature survey of public scRNA-seq datasets from mouse models of ischemic brain injury. This cross-study evaluation was constrained by inconsistent reporting conventions and a frequent lack of explicit quantification for *Gfap*⁺ reactive sub-populations, despite the central role of astrogliosis in stroke pathology. Furthermore, reported overall astrocyte yields fluctuated widely across studies (ranging from ~ 2% to > 20%, Fig. 1J, STab. 3). While diverse biological parameters (e.g., injury models, harvesting timepoint, and anatomical regions) undoubtedly contribute to this variance, these broad fluctuations highlight isolation workflows as an underappreciated source of technical heterogeneity across the single-cell field.

**Fig. 2:**
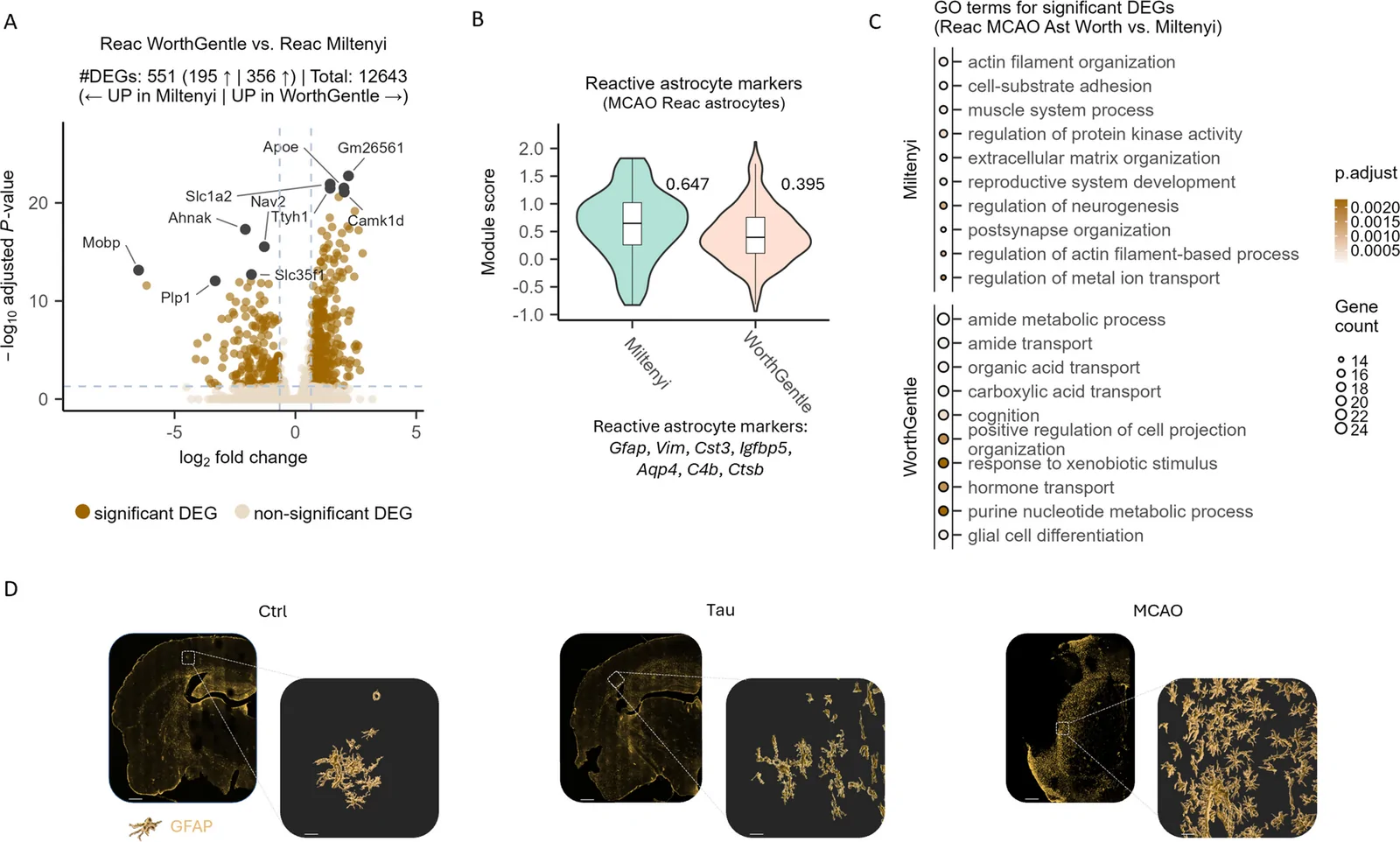
Protocol-dependent transcriptomic shifts in reactive astrocytes. **A** Volcano plot illustrating differential gene expression in Reac astrocytes isolated via WorthGentle (*n* = 398 cells) versus Miltenyi (*n* = 92 cells) protocols in MCAO (*N* = 8 mice total, *n* = 4 mice pooled per protocol). Each point represents a gene. **B** Violin plots with overlaid box plots showing module scores for reactive astrocyte markers isolated via WorthGentle (*n* = 398 cells) versus Miltenyi (*n* = 92 cells) protocols in MCAO (*N* = 8 mice total, *n* = 4 mice pooled per protocol). Box plots display the median (black line), the first and third quartiles (white box), and whiskers extending to 1.5 times the interquartile range. Median values are displayed in the top right corner. **C** Bubble plot visualizing the top 6 biological processes identified via GO analysis of the significant DEGs (from **A**) per protocol (*n* = 334 uniquely mapped input genes for WorthGentle, *n* = 190 uniquely mapped input genes for Miltenyi). **D** 2D and 3D immunohistochemical reconstructions of GFAP^+^ astrocytes in the studied pathologies. Scale bars: 500 µm (2D) and 20 µm (3D). *Abbreviations*: *Ctrl* control, *DEG* differentially expressed gene, *Hom* homeostatic, *GO* gene ontology, *MCAO* middle cerebral artery occlusion, *Miltenyi* commercial protocol Miltenyi Biotec, *ns* non-significant (*p* > 0.05), *Reac* reactive, *Tau* P301S tauopathy, *WorthGentle* optimized in-house protocol with minimal mechanical stress, *WorthMech* optimized in-house protocol with increased mechanical stress

Beyond differences in recovered proportions, we investigated whether isolation protocols alter the transcriptomic profile of surviving Reac astrocytes. Because the low number of Reac astrocytes recovered from the Tau model under the Miltenyi protocol precluded differential expression analysis (Fig. 1E, SFig. 2E), downstream transcriptomic and pathway comparisons were focused on the MCAO cohort. Pairwise comparison of MCAO Reac astrocytes isolated via WorthGentle versus Miltenyi revealed 551 candidate protocol-specific differentially expressed genes based on cell-level testing within the pooled cohorts (Fig. 2A, STab. 4). Canonical reactive astrocyte markers were detected across both isolation workflows (Fig. 2B); however, Reac astrocytes recovered via the WorthGentle method exhibited a marked enrichment in biological pathways governing cell projection organization and glial cell differentiation among the top enriched terms ranked by gene count (Fig. 2C, STab. 5). This transcriptomic profile aligns with reactive astrogliosis in vivo, where astrocytes undergo extensive morphological remodeling during glial scar formation, as visualized in our 3D immunohistochemical reconstructions (Fig. 2D). Because the structural remodeling of reactive astrocytes involves substantial rearrangement of the actin and intermediate filament cytoskeleton [13], we hypothesize that altered cellular rigidity could render these structurally complex cells particularly vulnerable to physical dissociation forces or selective loss during cell isolation [14, 15]. These observations indicate that the Miltenyi workflow results in a selective underrepresentation of reactive astrocytes characterized by morphological remodeling signatures, highlighting how workflow selection can act as an unintended filter on transcriptomic profiles. Our conclusions apply specifically to *Gfap*^high^ reactive astrocytes; whether *Gfap*^low^ or non-scar-forming states exhibit similar protocol-dependent vulnerability remains to be determined.

The implications for the broader neuro-genomics community are severe. Researchers routinely sequence unenriched, whole-tissue suspensions where overall astrocyte yields are inherently low. If protocols underrepresent Reac populations, these critical disease-driving cells may fall completely below downstream analytical thresholds. Consequently, researchers risk misinterpreting biological findings, overlooking vital therapeutic targets, and failing to capture the full spectrum of in vivo neuropathology. While our primary conclusions are established in the MCAO model (7 days post-injury), and the low tauopathy cell yield restricts the Tau findings to preliminary supporting observations, the central warning is clear: tissue dissociation is not a neutral preparatory step.

Several methodological constraints should be noted. First, because workflows differed across enzymatic cocktails, incubation times, and centrifugation forces (3000 × *g* debris pelleting vs. 60 × *g* gradient separation), cell loss cannot be isolated to a single biophysical or biochemical factor. Second, single-cell datasets relied on ACSA-2 enrichment, meaning these recovery patterns may not generalize directly to unenriched suspensions. Third, stroke evaluations were restricted to a single time point (7 days post-injury), leaving acute injury dynamics unexamined. Fourth, small animal cohorts and low reactive cell yield in the P301S tauopathy model restricted deeper sub-clustering and differential expression analysis. Finally, the literature survey remains purely observational, confounded by across-study differences in stroke models, anatomical margins, and isolation protocol.

## Conclusion

In conclusion, our study exposes a critical methodological vulnerability in single-cell transcriptomics: tissue dissociation protocols can underrepresent morphologically complex, reactive astrocyte populations. By demonstrating that a widely used commercial workflow underrepresents these reactive astrocytes and artificially skews their transcriptomic profiles—we highlight a pervasive risk of data misinterpretation across the field. To resolve this, we validated WorthGentle, an optimized protocol that improves the recovery of these disease-associated cellular states compared to the Miltenyi workflow. Our findings highlight that isolation pipelines operate as selective filters and underscore the necessity of workflow optimization when profiling fragile glial populations in neuropathology. As the neuro-genomics community continues to map the cellular drivers of acute brain injuries and chronic neurodegenerative diseases, these findings must serve our findings indicating that isolation parameters should be carefully considered when designing single-cell studies of reactive glial states.

## Methods

### Experimental Models

#### Middle Cerebral Artery Occlusion Model

C57BL/6J mice (male and female, 21–28 weeks old; Jackson Laboratory, Cat#000664) were housed in groups of 2–4 per temperature-controlled cage (20–24 °C) on a 12-h light/dark cycle with free access to food (ssniff V1124, ssniff Spezialdiäten GmbH) and water. Standard bedding (SAFE Select, J. Rettenmaier & Söhne), cellulose nesting material, and disposable cardboard tunnels were provided for enrichment. For the single-cell RNA-sequencing (scRNA-seq) experiment, 21 mice were distributed across three isolation protocols into permanent middle cerebral artery occlusion (MCAO; *n* = 4 mice per protocol: 2 females and 2 males per protocol) or control (*n* = 3 mice per protocol: 2 females and 1 male per protocol) groups (STab. 6). Ischemia was induced via distal left MCA occlusion using bipolar tweezers, and tissue was harvested after 7 days, as previously described [16].

#### Tauopathy Model

P301S transgenic mice [Tg(Thy1-MAPT*P301S)2541Godt], expressing the human 0N4R tau isoform with the P301S mutation under the control of the murine *Thy1* promoter (Tau; male and female, 22–26 weeks old) [17], were obtained from the University of Cambridge and bred in-house on a C57BL/6J genetic background. At 22–26 weeks of age, mice develop hyperphosphorylated tau filaments and reactive astrogliosis [17], which was verified in situ using GFAP confocal imaging (Fig. 2D). Eight mice were distributed across the three isolation protocols (*n* = 3 mice for Miltenyi: 2 females, 1 male; *n* = 3 mice for WorthGentle: 2 females, 1 male; *n* = 2 mice for WorthMech: 1 female, 1 male; STab. 6). Animals were housed in individually ventilated cages (Green line Tecniplast) under standard temperature (20–24 °C) and light cycle conditions, with free access to standard diet (Altromin 1410 formula, Cat#1414) and filtered chlorinated water.

### Isolation of Cortex Tissue

Mice were anesthetized with sodium pentobarbital (100 mg/kg, i.p.; Sigma-Aldrich, Cat#P3761; dissolved in 0.9% saline) and transcardially perfused with cold (4–8 °C) isolation buffer (IB: 136.0 mM NaCl [Sigma-Aldrich, Cat#S9888], 5.4 mM KCl [Sigma-Aldrich, Cat#P5405], 10.0 mM HEPES [Sigma-Aldrich, Cat#H3375], 5.5 mM glucose [Sigma-Aldrich, Cat#G8270]; pH 7.4, 290 ± 3 mOsmol/kg). Control, MCAO, or Tau parietal cortex segments (+ 2 mm to −2 mm from bregma) were dissected from the left hemisphere, explicitly excluding ventral white matter. To minimize technical batch skews and eliminate systematic differences in operator handling, specialized procedures were executed by single designated personnel: surgical distal MCAO occlusions (~ 20 min per animal) were carried out by a single dedicated surgeon, transcardial perfusions and rapid whole-brain isolations were performed strictly by the same investigator in < 5 min per mouse, and coronal cortical microdissections were completed by a single experienced operator in < 2 min per brain. Animals sharing the same date of birth from synchronized breeding cohorts were evenly distributed across the three dissociation workflows to balance biological background across processing days (STab. 6).

### Dissociation Protocols

#### Commercial Protocol (Miltenyi)

Brain tissue was enzymatically and mechanically dissociated using the Adult Brain Dissociation Kit (Miltenyi Biotec, Cat#130-107-677) according to the manufacturer’s protocol. Tissue was processed in gentleMACS C Tubes (Miltenyi Biotec, Cat#130-093-237) on a gentleMACS Octo Dissociator with Heaters using the 37C_ABDK_01 program. The suspension was passed through a 50 µm CellTrics filter (Sysmex, Cat#04-004-2327). Debris and erythrocytes were removed using the manufacturer’s Debris Removal Solution and Red Blood Cell Removal Solution prior to magnetic labeling with the Anti-ACSA-2 MicroBead Kit (Miltenyi Biotec, Cat#130-097-679).

#### Optimized Low-Shear in-House Protocol (WorthGentle)

Dissected mouse brain cortices were placed in 1 mL of papain solution (20 U/mL papain [Worthington Biochemical Corp., Cat#LK003176], 95 U/mL DNase I [Worthington Biochemical Corp., Cat#LK003170], 23.8 µg/mL Actinomycin D [ActD; Sigma-Aldrich, Cat#A1410-10MG; dissolved in dimethyl sulfoxide [Sigma-Aldrich, Cat#472301-100ML]; dissolved in sterile Earle’s balanced salt solution [EBSS, Worthington Biochemical Corp., Cat#LK003188]) and mechanically pre-processed using a tube pestle (Thermo Fisher, Cat#12141364). Samples were incubated at 37 °C for 60 min with shaking (ThermoMixer C, Eppendorf) at 800 rpm with periodic inversion, followed by gentle homogenization with a wide-bore P1000 tip (Axygen, Cat#TF­1005-WB-R-S).

After centrifugation (300 × *g*, 5 min, 4 °C, break 1, acceleration 1; Centrifuge 5804R, Eppendorf), the pellet was digested with 950 µL of pre-warmed Trypsin solution (containing 0.05% of Trypsin from porcine pancreas [Sigma-Aldrich, Cat #T7409], 0.53 µM EDTA [Sigma-Aldrich, Cat#E7889-100ML], 25 µg/mL ActD; dissolved in Dulbecco′s Phosphate Buffered Saline [EMD Millipore, Cat#D8537-500ML]) at 37 °C for 5 min, disrupted with a 1 mL wide-bore tip, and quenched in 2 mL of STOP solution (0.2 mg/mL of ovomucoid inhibitor and 0.2 mg/mL of bovine serum albumin [BSA; Worthington Biochemical Corp., Cat#LK003182], 2.5 µg/mL ActD; dissolved in EBSS).

The suspension was spun (300 × *g*, 5 min, 4 °C, break 3, acceleration 3), gently resuspended with a standard P1000 tip (Axygen, Cat#TF-1000-L-R-S) in 600 µL of resuspension solution [1 mg/mL of ovomucoid protease inhibitor and 1 mg/mL of BSA, 14 U Deoxyribonuclease I; dissolved in EBSS, and ActD (2.5 µg/mL)], filtered through a 50 µm CellTrics filter, and carefully layered over a 1 mL ovomucoid protease inhibitor solution (1 mg/mL of ovomucoid protease inhibitor and 1 mg/mL of BSA; dissolved in EBSS), and centrifuged (60 × *g*, 6 min, 4 °C, break 0, acceleration 0). The cell pellet was resuspended in 1 mL of preservation buffer (PB; 1% BSA [Gibco, Cat#15260037], 2.5 µg/mL of ActD; dissolved in IB) using a wide-bore P1000 tip.

Myelin was depleted using Myelin Removal Beads II (Miltenyi Biotec, Cat# 130-096-733) over an LS column fixed to a QuadroMACS separator. For myelin depletion, the cell pellet was resuspended to 360 µL in cold PB solution and 40 µL Myelin Removal Beads II, and incubated on ice for 5 min. Cells were washed with 4 mL of PB solution with a wide-bore P1000 tip and centrifuged (300 × *g*, 5 min, 4 °C, break 3, acceleration 3). The pellet was resuspended in 1 mL of PB solution with a wide-bore P1000 tip and passed through a pre-equilibrated LS column (Miltenyi Biotec, Cat# 130-042-401) with 3 mL PB wash on a QuadroMACS magnet (Miltenyi Biotec, Cat#130-090-976).

Astrocytes were positively selected from the myelin-depleted fraction using the Anti­ACSA-2 MicroBead Kit. The collected cells were centrifuged (300 × *g*, 5 min, 4 °C, break 3, acceleration 3) and the cell pellet was resuspended in 10 µL of MicroBeads (200 µL wide-bore tip), and incubated at 4 °C for 15 min, mixing every 5 min. Labeled cells were washed with 1 mL of AstroMACS Separation Buffer (Miltenyi Biotec, Cat#130-117-336) with a wide-bore P1000 tip and centrifuged (300 × *g*, 5 min, 4 °C, break 3, acceleration 3). The pellet was resuspended in 500 µL of AstroMACS Separation Buffer with a standard P1000 tip and loaded onto a pre-equilibrated MS column (Miltenyi Biotec, Cat#130-042-201) on an OctoMACS magnet (Miltenyi Biotec, #130-042-109). After four washes with 500 µL of AstroMACS Separation Buffer, the column was uncoupled from the magnet, and astrocytes were eluted by flushing twice with 1 mL of AstroMACS Separation Buffer using the column plunger. A detailed step-by-step protocol is available at Protocols.io [18].

#### Optimized in-House Protocol with Increased Shear Stress (WorthMech)

This protocol was identical to WorthGentle, except tissue suspensions during papain incubation were mechanically disrupted by pipetting 5 times with a standard P1000 tip every 10 min instead of periodic inversion. Standard tips were systematically used in all subsequent processing, washing, and resuspension steps in place of wide-bore tips.

### Library Preparation and Sequencing

Cell concentrations were measured on a DeNovix CellDrop counter. Cells were fixed using the Evercode Cell Fixation v3 Kit (Parse Biosciences, Cat#ECFC3300) and stored at − 80 °C. Single-cell suspensions from each biological condition and isolation protocol were fixed using the Evercode Cell Fixation v3 Kit (Parse Biosciences, Cat#ECFC3300) (STab.6), targeting ~ 2000 cells per experimental condition across 9 conditions (3 isolation protocols × 3 biological models; 18,000 target cells total). All fixed samples were run through three sequential rounds of split-pool combinatorial barcoding using the Evercode WT v3 Kit (Parse Biosciences, Cat#ECWT3300). The resulting pool was partitioned into 10 sublibraries to optimize cDNA amplification, followed by simultaneous sequencing of all sublibraries in a single sequencing run on an Illumina NovaSeq X Plus instrument using a 25B flow cell and the NovaSeq X Series 25B Reagent Kit (Illumina, Cat#20104706).

### scRNA-seq Data Processing and Clustering

Reads were mapped to the GRCm38 mouse genome (gencode_vM8) via the spipe pipeline (v1.3.1). To distinguish authentic single-cell transcriptomes from ambient background RNA, cell calling was performed using the emptyDrops from the DropletUtils package (v1.14.1) [19]. Barcodes with a baseline total unique molecular identifier count ≥ 100 were evaluated, and cells were retained at an adjusted false discovery rate ≤ 0.001. Quality control filtering in Seurat (v5.0.1) [20] removed low-quality barcodes with ≤ 500 detected genes or ≥ 20% mitochondrial transcripts. Multicellular doublets were predicted and eliminated on a per-sample basis using DoubletFinder (v2.0.4) [21] with an expected 5% doublet formation rate based on standard loading densities. Sequential cell counts retained at each filtering step for every condition are provided in STab. 7.

Filtered count matrices were normalized using SCTransform, regressing out the percentage of mitochondrial transcripts. Because all samples were co-barcoded and sequenced in a single sequencing pool, data were merged without non-linear batch integration algorithms to preserve genuine protocol-driven population differences. Dimensionality reduction was performed using principal component analysis (PCA), and the first 20 principal components were used for graph-based clustering (FindNeighbors and FindClusters; resolution 0.2, random seed = 42) to distinguish astrocytes from contaminating non-astrocytic cells. Next, the identified astrocyte cluster was subsetted, and from these cells, only *Aldh1l1*-positive cells (*Aldh1l1* > 0 count) were retained to eliminate potential non-astrocytic cells. This filtering step excluded an average of 15.11% of cells across workflows (15.23% in Miltenyi, 13.81% in WorthGentle, and 16.29% in WorthMech), yielding a final curated baseline of *n* = 8586 astrocytes. The curated astrocyte population was then re-normalized via SCTransform and re-clustered at a resolution of 0.075.

### Identification of Cluster Markers

Marker genes for each cell population were identified using the FindAllMarkers function in Seurat, based on the default Wilcoxon rank-sum test. Differentially expressed genes were considered significant markers if they exhibited a log-fold change threshold > 0.65 and were detected in at least 80% of cells within a given cluster. Unsupervised clustering of the curated astrocyte population was performed across all conditions and workflows simultaneously without knowledge of protocol identity. Astrocyte sub-clusters were annotated as homeostatic or Reac based on data-driven differential expression across all genes (STab. 2) and corroborated by projecting published multi-gene reactive and disease-associated astrocyte signatures [7].

Overlap between cluster marker genes and defined gene sets was evaluated using Seurat’s AddModuleScore function. To assess transcriptomic similarities, the identified astrocyte clusters were evaluated against the signature gene sets from Habib et al. [7] and visualized on UMAP embeddings using FeaturePlot. Furthermore, AddModuleScore was used to quantify transcriptomic similarities in reactive astrocyte signatures across isolation protocols (Miltenyi vs. WorthGentle), with results visualized using violin plots.

### Differential Gene Expression Analysis

Differential gene expression analysis was performed to identify upregulated and downregulated genes in Reac astrocytes from the MCAO model, comparing the WorthGentle and Miltenyi isolation protocols. Pairwise comparisons were conducted using Seurat’s FindMarkers function, applying the default Wilcoxon rank-sum test to the normalized RNA assay data. Genes were considered significantly differentially expressed if they were detected in at least 10% of the cells in either comparison group, exhibiting an absolute log2 fold change > 0.65 and a Bonferroni-adjusted p-value < 0.05 across all genes in the dataset (STab. 4). Because cortical cell suspensions were pooled across biological animals prior to ACSA-2 magnetic enrichment and Parse Evercode fixation without individual sample-level cell hashing, single cells could not be computationally demultiplexed back to individual donor mice. Consequently, pseudobulk aggregation or animal-level mixed-effects models could not be applied. Differentially expressed gene identification was therefore performed using cell-level non-parametric testing across pooled cohorts, and the resulting gene list should be interpreted as candidate protocol-associated transcriptomic shifts.

### Gene Ontology Enrichment Analysis

Functional annotation was performed via gene ontology enrichment analysis using the compareCluster function from the clusterProfiler package (v4.14.4) [22], querying the Gene Ontology Biological Process domain. Analysis was referenced against the background of 12,643 detectably expressed genes evaluated during pairwise differential expression analysis. Target genes were evaluated using Ensembl identifiers (stripped of version suffixes) mapped via the Bioconductor annotation database org.Mm.eg.db. Genes were excluded from this testing if they lacked an annotated Entrez ID in org.Mm.eg.db or had no assigned Biological Process terms. Multiple testing correction was performed using the Benjamini–Hochberg false discovery rate procedure (requiring adjusted *p* < 0.05 and *q* < 0.05). Redundant parent and descendant terms were filtered using clusterProfiler::simplify(), and Ensembl IDs were translated to official gene symbols for tabular presentation via clusterProfiler::setReadable() (STab. 5).

### Spatial Transcriptomics and Public Datasets

Dissociation-free spatial transcriptomics data were obtained from the publicly available 10 × Genomics Visium dataset [11]. While our scRNA-seq and sc-qPCR experiments specifically analyzed cortex segments (+ 2 mm to –2 mm relative to bregma), the spatial transcriptomics analysis utilized whole coronal hemispheric sections. To maintain anatomical consistency across platforms, in silico spatial subsetting was restricted strictly to the cerebral cortex and the ischemic lesion core. The spatial dataset matched our single-cell cohort in mouse strain (C57BL/6J), injury model, and post-injury time point (7 days post-injury). Spots covering the cortex and ischemic lesion were filtered for pan-astrocytic identity (*Aldh1l1* > 0). A reference baseline was defined as the mean normalized *Gfap* expression across all *Gfap*⁺ (*Gfap* > 0) spots within the uninjured control cortex. Spots exceeding this control mean threshold were classified as *Gfap*^high+^ reactive astrocyte spots.

For a literature survey, reported astrocyte yields and *Gfap* expression frequencies were compiled from published independent mouse scRNA-seq studies of ischemic brain injury. Inclusion was restricted to studies meeting two explicit criteria: (1) use of an in vivo mouse model of ischemic stroke, and (2) single-cell transcriptomic profiling processed (excluding bulk or single-nucleus assays). Biological and technical metadata—including age, injury model, time point, dissociation enzyme, brain region, proportion of astrocytes and *Gfap*^+^ cells —were cataloged in (STab. 3) to illustrate the breadth of reporting conventions across the literature.

### Immunohistochemistry

For immunohistochemical analyses, animals were deeply anesthetized with sodium pentobarbital (100 mg/kg, i.p.) and perfused transcardially with 20 mL of saline followed by 20 mL of cooled 4% paraformaldehyde (Sigma-Aldrich, Cat#P6148) in 0.1 M phosphate buffer (154.8 mM Na2HPO4 [Sigma-Aldrich, Cat#S0876] and 45.2 mM NaH2PO4 [Sigma-Aldrich, Cat#S5011], dissolved in distilled water; pH 7.4). Brains were carefully dissected, post-fixed in 4% paraformaldehyde for 3 h at 4 °C, and cryoprotected through a sucrose gradient (10% to 30% sucrose; Lach-ner, Cat#40135-AP0). Coronal sections (30 μm thick) were cut using a cryostat (Leica CM1950, Leica Microsystems).

For immunohistochemical staining, tissue sections were washed in phosphate-buffered saline and incubated in a blocking solution consisting of 5% ChemiBLOCKER (Millipore, Cat#2170) and 0.2% Triton x-100 (Sigma-Aldrich, Cat#9036-19-5) in Dulbecco’s Phosphate Buffered Saline to prevent non-specific binding. The blocking solution was also used as the diluent for the antisera. Sections were incubated overnight at 4–8 °C with primary antibody against GFAP (mouse anti-GFAP monoclonal antibody, 1:500, unconjugated, clone G-A-5, Sigma-Aldrich, Cat#G3893, RRID: AB_477010). After washing, sections were incubated for 2 h at 4–8 °C with fluorophore-conjugated secondary antibody (donkey anti-mouse IgG (H + L) highly cross-adsorbed secondary antibody, Alexa Fluor™ Plus 488, Thermo Fisher Scientific, Cat# A32766TR, RRID: AB_2866493).

Confocal imaging was performed using an Andor Dragonfly 503 spinning disk confocal microscope equipped with a 20 × water objective. Hemispheric tile scans (15 × 15) were acquired as stacks with 2 μm step intervals. Automated tile stitching with a 10% overlap was executed using Fusion software (Oxford Instruments). Maximum intensity z-projection images were generated using Imaris (Oxford Instruments). For representative structural visualizations, three-dimensional reconstructions of individual astrocytes were created using the Surface rendering module in Imaris.

### Single-Cell Quantitative PCR

#### Cell Isolation and Fluorescence-Activated Cell Sorting

The cells were isolated as described previously (see Dissociation of brain tissue into: A standardized commercial protocol (Miltenyi) and Dissociation of brain tissue into: Optimized in-house protocol with minimized shear stress (WorthGentle)). For this sc­qPCR experiment, 0.5 µL of Calcein Green (1:1000 dilution, Invitrogen, Cat#C34852) was added during the ACSA-2 MicroBead labeling step. The resulting ACSA-2-enriched single-cell suspension was prepared for sorting by adding 1 µL of Hoechst 33258 (Sigma-Aldrich, Cat#94403) directly to the suspension.

Calcein Green^+^Hoechst 33258^−^ single cells were isolated via fluorescence-activated cell sorting into 384-well plates (Bio-Rad, Cat#HSP3801) employing a BD FACSMelody system (BD FACSChorus v1.3 software, BD FACS Aria Fusion, BD Biosciences) equipped with a 100-μm nozzle, with sample and plate cooling maintained at 4 °C and index sorting enabled (SFig. 3 A, B). Each well in 384-well plates pre-contained 0.3 µL of lysis buffer—consisting of 0.5 mM dNTPs (Thermo Scientific, Cat#R0192), 0.125 µM oligo dTVN30 (IDT), 5% PEG8000 (Sigma-Aldrich, Cat#P1458-25ML), 0.0125 µL SIRV4 diluted 1:2000 (Lexogen, Cat#141.01), and 0.2 U SEQURNA Thermostable RNase Inhibitor (SEQURNA AB, Cat#SQ00203) diluted in UltraPure DNase/RNase-Free Distilled Water (NFW, Invitrogen, Cat#10977035), dispensed into each well alongside 3 µL of silicone oil (100 cSt, Sigma-Aldrich, Cat#378364-250ML) using the MANTIS® Liquid Dispenser (FORMULATRIX), inspired by Hagemann-Jensen et al. [23]. After sorting, the plates were centrifuged and stored at − 80 °C.

#### Quantitative PCR

The stored 384-well plates were thawed and immediately incubated at 72 °C for 10 min. Next, 0.1 µL of the RT master mix (25 mM Tris–HCl [pH 8.0; Invitrogen, Cat#AM9856], 2.5 mM MgCl₂ [Invitrogen, Cat#AM9530G], 30 mM NaCl [Invitrogen, Cat#AM9760G], 1 mM GTP [Thermo Scientific, Cat#R1461], 8 mM USB Dithiothreitol [Thermo Scientific, Cat#707265ML], 0.75 µM Smart-seq3xpress Template Switching Oligo [IDT], and 2 U/µL of Maxima H-minus Reverse Transcriptase [Thermo Scientific, Cat#EP0753]) was dispensed into each well using the MANTIS® Liquid Dispenser. Reverse transcription was executed on a C1000 Touch Thermal Cycler (Bio-Rad) at 42 °C for 90 min, followed by 10 template-switching cycles consisting of 50 °C for 2 min and 42 °C for 2 min. The enzyme was subsequently heat-inactivated at 85 °C for 5 min. Immediately following reverse transcription, cDNA pre-amplification was performed using the following cycling program: 95 °C for 1 min, followed by 15 cycles of 98 °C for 10 s, 65 °C for 30 s, and 68 °C for 4 min, with a final extension at 72 °C for 10 min. Post–pre-amplification, the cDNA was diluted with 9 µL of NFW and stored at − 20 °C overnight.

For downstream verification, 1 µL of the pre-amplified cDNA was diluted in 13 µL of NFW. A 1 µL aliquot of this diluted cDNA framework was then combined with 2 µL of a qPCR master mix, which comprised 1.5 µL of TATAA SYBR GrandMaster Mix (TATAA Biocenter AB, Cat#TA01­5000), 0.38 µL NFW and 0.12 µL primer mix (10 µM forward [FW] and 10 µM reverse [RV] primers). The specific primer sequence (5′ → 3′) utilized were: *Actb*–FW GCTCCTAGCACCATGAAGAT, RV–TAAAACGCAGCTCAGTAACAG (Invitrogen); *Aldh1l1*–FW CTGGAAGATGGCAAGATGATG, RV–TTCTACCTCTGGGACATTGG (Invitrogen); *Gfap*–FW ATTCGCACTCAATACGAGGC, RV–AGGTCTGCAAACTTAGACCG (Invitrogen).

Quantitative PCR was executed on a thermal cycler using a total reaction volume of 3 µL. The thermal cycling program initiated with a denaturation step at 95 °C for 3 min, followed by 45 amplification cycles consisting of denaturation at 95 °C for 15 s, primer annealing at 60 °C for 20 s, and elongation at 72 °C for 20 s. To evaluate product specificity and identify non-specific amplification, a post-amplification melt curve analysis was performed by holding at 65 °C for 5 s, followed by a gradual temperature ramp up to 95 °C at an incremental rate of 0.5 °C every 5 s.

#### Data Analysis

The quality filtering of raw 96-well qPCR data per animal was performed in R to evaluate astrocyte marker distributions. Wells were retained for population enumeration only if they met threshold of Cq ≤ 30 along with specific melting temperature profiles (*Actb*: 83–85 °C; *Aldh1l1*: 83–85 °C; *Gfap*: 78–79 °C). The relative abundance of reactive astrocytes was defined as the percentage of *Actb*⁺*Aldh1l1*⁺*Gfap*⁺ wells out of the total pan-astrocyte baseline (*Actb*⁺*Aldh1l1*⁺). Protocol-dependent differences in reactive astrocyte capture efficiency between workflows were evaluated using a two-tailed Welch’s two-sample *t*-test, reporting the exact difference in group means (ΔMean) and its associated 95% confidence interval (95% CI). Standardized effect sizes were quantified by calculating Cohen’s *d* using the rstatix package (v0.7.3) in R.

## Supplementary Information

Below is the link to the electronic supplementary material.

ESM 1 (ZIP 1.35 MB)

## Data Availability

The datasets generated and analyzed during the current study are available in the NCBI repository (https://www.ncbi.nlm.nih.gov/geo/query/acc.cgi?acc=GSE339378) and Zenodo repository (https://zenodo.org/records/20339679). The code for analysis is available on GitHub (https://github.com/GliaOmicsLab/astrocyte-isolation-scRNAseq). A detailed version of the optimized dissociation protocol is available at Protocol.io (https://www.protocols.io/private/E6D2C6A7897011F0B7560A58A9FEAC02).

## Abbreviations

CNS: Central nervous system
FW: Forward
GFAP: Glial fibrillary acidic protein
MCAO: Middle cerebral artery occlusion
Miltenyi: Commercial dissociation protocol by Miltenyi Biotec
Reac: Reactive
RV: Reserve
sc-qPCR: Single-cell quantitative polymerase chain reaction
scRNA-seq: Single-cell RNA sequencing
Tau: P301S tauopathy
WorthGentle: Optimized in-house protocol with minimal mechanical shear stress
WorthMech: Modified in-house protocol with increased mechanical shear stress

## References

[1] Liddelow SA, Guttenplan KA, Clarke LE, Bennett FC, Bohlen CJ, Schirmer L et al (2017) Neurotoxic reactive astrocytes are induced by activated microglia. Nature 541:481–487. 10.1038/nature21029 PMC5404890 28099414

[2] Kamphuis W, Mamber C, Moeton M, Kooijman L, Sluijs JA, Jansen AHP et al (2012) GFAP isoforms in adult mouse brain with a focus on neurogenic astrocytes and reactive astrogliosis in mouse models of Alzheimer disease. PLoS One 7:e42823. 10.1371/journal.pone.0042823 PMC3418292 22912745

[3] Li H, Zhang N, Lin H-Y, Yu Y, Cai Q-Y, Ma L et al (2014) Histological, cellular and behavioral assessments of stroke outcomes after photothrombosis-induced ischemia in adult mice. BMC Neurosci 15:58. 10.1186/1471-2202-15-58 PMC4039545 24886391

[4] Madissoon E, Wilbrey-Clark A, Miragaia RJ, Saeb-Parsy K, Mahbubani KT, Georgakopoulos N et al (2020) scRNA-seq assessment of the human lung, spleen, and esophagus tissue stability after cold preservation. Genome Biol 21:1. 10.1186/s13059-019-1906-x PMC6937944 31892341

[5] O’Flanagan CH, Campbell KR, Zhang AW, Kabeer F, Lim JLP, Biele J et al (2019) Dissociation of solid tumor tissues with cold active protease for single-cell RNA-seq minimizes conserved collagenase-associated stress responses. Genome Biol 20:210. 10.1186/s13059-019-1830-0 PMC6796327 31623682

[6] Denisenko E, Guo BB, Jones M, Hou R, De Kock L, Lassmann T et al (2020) Systematic assessment of tissue dissociation and storage biases in single-cell and single-nucleus RNA-seq workflows. Genome Biol 21:130. 10.1186/s13059-020-02048-6 PMC7265231 32487174

[7] Habib N, McCabe C, Medina S, Varshavsky M, Kitsberg D, Dvir-Szternfeld R et al (2020) Disease-associated astrocytes in Alzheimer’s disease and aging. Nat Neurosci 23:701–706. 10.1038/s41593-020-0624-8 PMC9262034 32341542

[8] Delahaye M, Lawrence K, Ward SJ, Hoare M (2015) An ultra scale‐down analysis of the recovery by dead‐end centrifugation of human cells for therapy. Biotechnology and Bioengineering 112:997–1011. 10.1002/bit.25519 PMC4402021 25545057

[9] Batiuk MY, De Vin F, Duqué SI, Li C, Saito T, Saido T et al (2017) An immunoaffinity-based method for isolating ultrapure adult astrocytes based on ATP1B2 targeting by the ACSA-2 antibody. J Biol Chem 292:8874–91. 10.1074/jbc.M116.765313 PMC5448122 28373281

[10] Kantzer CG, Boutin C, Herzig ID, Wittwer C, Reiß S, Tiveron MC et al (2017) Anti-ACSA-2 defines a novel monoclonal antibody for prospective isolation of living neonatal and adult astrocytes. Glia 65:990–1004. 10.1002/glia.23140 28317180

[11] Zucha D, Abaffy P, Kirdajova D, Jirak D, Kubista M, Anderova M et al (2024) Spatiotemporal transcriptomic map of glial cell response in a mouse model of acute brain ischemia. Proc Natl Acad Sci U S A 121:e2404203121. 10.1073/pnas.2404203121 PMC11573666 39499634

[12] Rosenberg AB, Roco CM, Muscat RA, Kuchina A, Sample P, Yao Z et al (2018) Single-cell profiling of the developing mouse brain and spinal cord with split-pool barcoding. Science 360:176–182. 10.1126/science.aam8999 PMC7643870 29545511

[13] Murk K, Blanco Suarez EM, Cockbill LMR, Banks P, Hanley JG (2013) The antagonistic modulation of Arp2/3 activity by N-WASP/WAVE2 and PICK1 defines dynamic changes in astrocyte morphology. J Cell Sci. 10.1242/jcs.125146 PMC3757329 23843614

[14] Lee S-M, Nguyen T-H, Na K, Cho I-J, Woo DH, Oh J-E et al (2015) Nanomechanical measurement of astrocyte stiffness correlated with cytoskeletal maturation: nanomechanical measurement of astrocyte stiffness. J Biomed Mater Res 103:365–370. 10.1002/jbm.a.35174 24665040

[15] Curry N, Ghézali G, Kaminski Schierle GS, Rouach N, Kaminski CF (2017) Correlative STED and atomic force microscopy on live astrocytes reveals plasticity of cytoskeletal structure and membrane physical properties during polarized migration. Front Cell Neurosci 11:104. 10.3389/fncel.2017.00104 PMC5396045 28469559

[16] Dostalova D, Abaffy P, Rohlova E, Kriska J, Knotek T, Tureckova J et al (2026) Adapted Smart-seq3xpress facilitates selective microglial transcriptomic profiling from frozen brain tissue. Cell Mol Neurobiol 46:113. 10.1007/s10571-026-01743-5 PMC13357468 42133140

[17] Allen B, Ingram E, Takao M, Smith MJ, Jakes R, Virdee K et al (2002) Abundant Tau filaments and nonapoptotic neurodegeneration in transgenic mice expressing human P301S Tau protein. J Neurosci 22:9340–51. 10.1523/JNEUROSCI.22-21-09340.2002 PMC6758022 12417659

[18] Dostalova D, Abaffy P, Valihrach L (2026) Single-cell astrocyte isolation to maximize Gfap+ cell recovery. protocols.io. 10.17504/protocols.io.x54v97yemg3e/v1

[19] Lun ATL, Riesenfeld S, Andrews T, Dao TP, Gomes T, Marioni JC (2019) EmptyDrops: distinguishing cells from empty droplets in droplet-based single-cell RNA sequencing data. Genome Biol 20:63. 10.1186/s13059-019-1662-y PMC6431044 30902100

[20] Hao Y, Hao S, Andersen-Nissen E, Mauck WM, Zheng S, Butler A et al (2021) Integrated analysis of multimodal single-cell data. Cell 184:3573-3587.e29. 10.1016/j.cell.2021.04.048 PMC8238499 34062119

[21] McGinnis CS, Murrow LM, Gartner ZJ (2019) DoubletFinder: doublet detection in single-cell RNA sequencing data using artificial nearest neighbors. Cell Syst 8:329-337.e4. 10.1016/j.cels.2019.03.003 PMC6853612 30954475

[22] Wu T, Hu E, Xu S, Chen M, Guo P, Dai Z et al (2021) clusterProfiler 4.0: a universal enrichment tool for interpreting omics data. Innovation (Cambridge (Mass)) 2:100141. 10.1016/j.xinn.2021.100141 PMC8454663 34557778

[23] Hagemann-Jensen M, Ziegenhain C, Sandberg R (2022) Scalable single-cell RNA sequencing from full transcripts with Smart-seq3xpress. Nat Biotechnol 29:1452–7. 10.1038/s41587-022-01311-4 PMC9546772 35637418

