# Supplementary material for: Tissue Dissociation Workflows Influence Reactive Astrocyte Recovery in Single-Cell Transcriptomics
Source: Mol Neurobiol. 2026 Oct 16;63(1):974. doi: 10.1007/s12035-026-06278-w (PMC13675257; doi:10.1007/s12035-026-06278-w)
Supplement: Supplementary file 1 [file 12035_2026_6278_MOESM1_ESM.zip › SFig. 1-3.pdf]

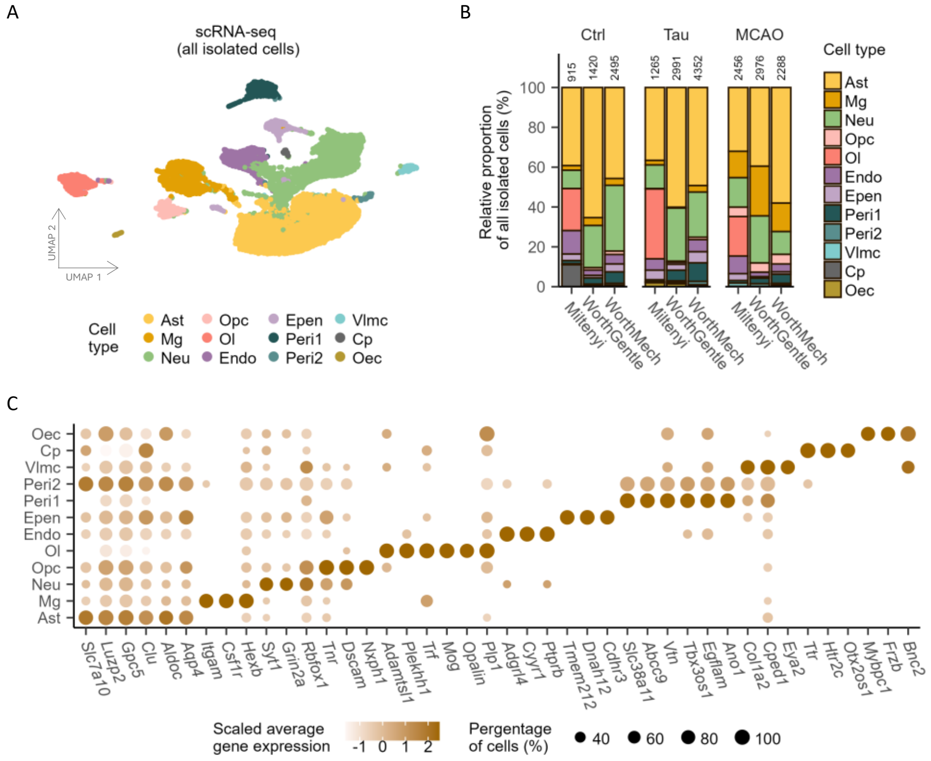

SFig. 1: Characterization of cells obtained in the scRNA-seq dataset. (A) UMAP projection of all cell populations detected among the sorted ACSA-2+ cells across all experimental conditions (*N* = 29 mice total, *n* = 21,158 cells). **(B)** Stacked bar plot showing the proportional frequency of detected populations, with the absolute cell counts for each condition displayed at the top of the respective bars (*N* = 29 mice total, *n* = 3 mice pooled per protocol for Ctrl and Tau, except for Tau WorthMech with *n* = 2 mice; *n* = 4 mice pooled per protocol for MCAO). **(C)** Dot plot illustrating the expression of marker genes used to identify the detected cell populations.

Abbreviations: Ast – astrocytes, Cp – choroid plexus, Ctrl – control, Endo – endothelial cells, Epen – ependymal cells, MCAO – middle cerebral artery occlusion, Mg – microglia, Miltenyi – commercial protocol Miltenyi Biotec, Neu – neurons, Oec – olfactory ensheathing cells, Ol – oligodendrocytes, Opc – oligodendrocyte progenitor cells, Peri 1/2 – pericytes 1 or 2, Tau – P301S tauopathy, Vlmc – vascular leptomeningeal cells, WorthGentle – optimized in-house protocol with minimal mechanical stress, WorthMech – optimized in-house protocol with increased mechanical stress.

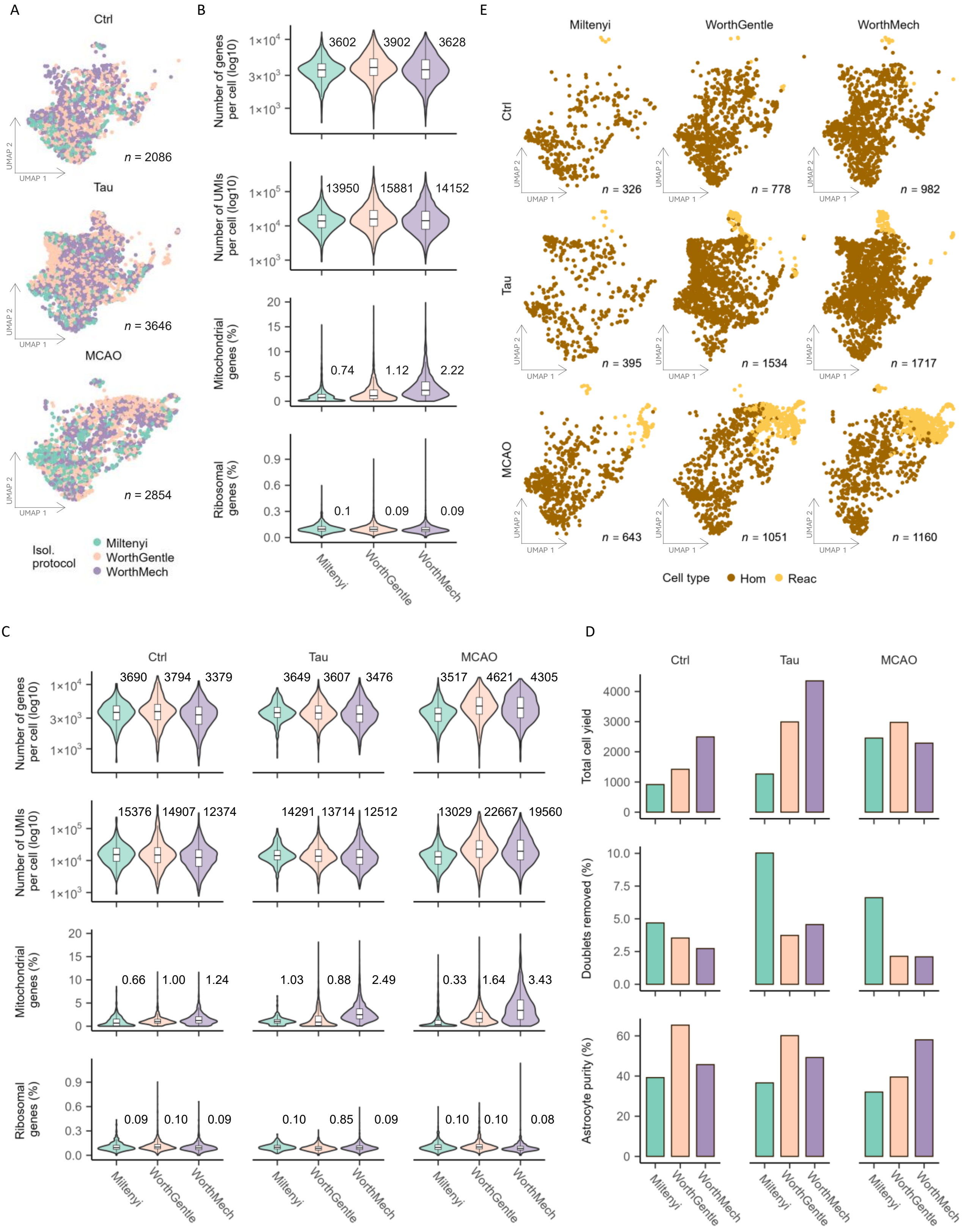

SFig. 2: The proportion of reactive astrocytes in scRNA-seq datasets is affected by isolation protocols. (A) UMAP projection of the astrocyte population, segregated by pathology (*N* = 29 mice total). **(B)** Violin plots with overlaid box plots displaying quality control metrics across the three isolation protocols (*n* = 8,586 cells). Box plots display the median (black line), the first and third quartiles (white box), and whiskers extending to 1.5 times the interquartile range. Median values are displayed in the top right corner. **(C)** Violin plots with overlaid box plots displaying quality control metrics across the three isolation protocols presented per tested condition (n = 8,586 astrocytes). Box plots display the median (black line), the first and third quartiles (white box), and whiskers extending to 1.5 times the interquartile range. Median values are displayed in the top right corner. **(D)** Bar plots showing the total number of quality-filtered single cells recovered per condition across all captured brain cell types (total cell yield), proportion of high-confidence cell doublets identified and eliminated prior to downstream analysis using DoubletFinder (doublets removed (%)) and proportion of validated *Aldh1l1*$^{+}$ astrocytes relative to the total recovered cells per condition (astrocyte purity (%)), **(E)** UMAP projection of the astrocyte population, segregated by both isolation protocol and pathology (*N* = 29 mice total, *n* = 3 mice pooled per protocol for Ctrl and Tau, except for Tau WorthMech with *n* = 2 mice; *n* = 4 mice pooled per protocol for MCAO).

Abbreviations: Ctrl – control, Hom – homeostatic, MCAO – middle cerebral artery occlusion, Miltenyi – commercial protocol Miltenyi Biotec, n – number of cells, Reac – reactive, Tau – P301S tauopathy, WorthGentle – optimized in-house protocol with minimal mechanical stress, WorthMech – optimized in-house protocol with increased mechanical stress.

A

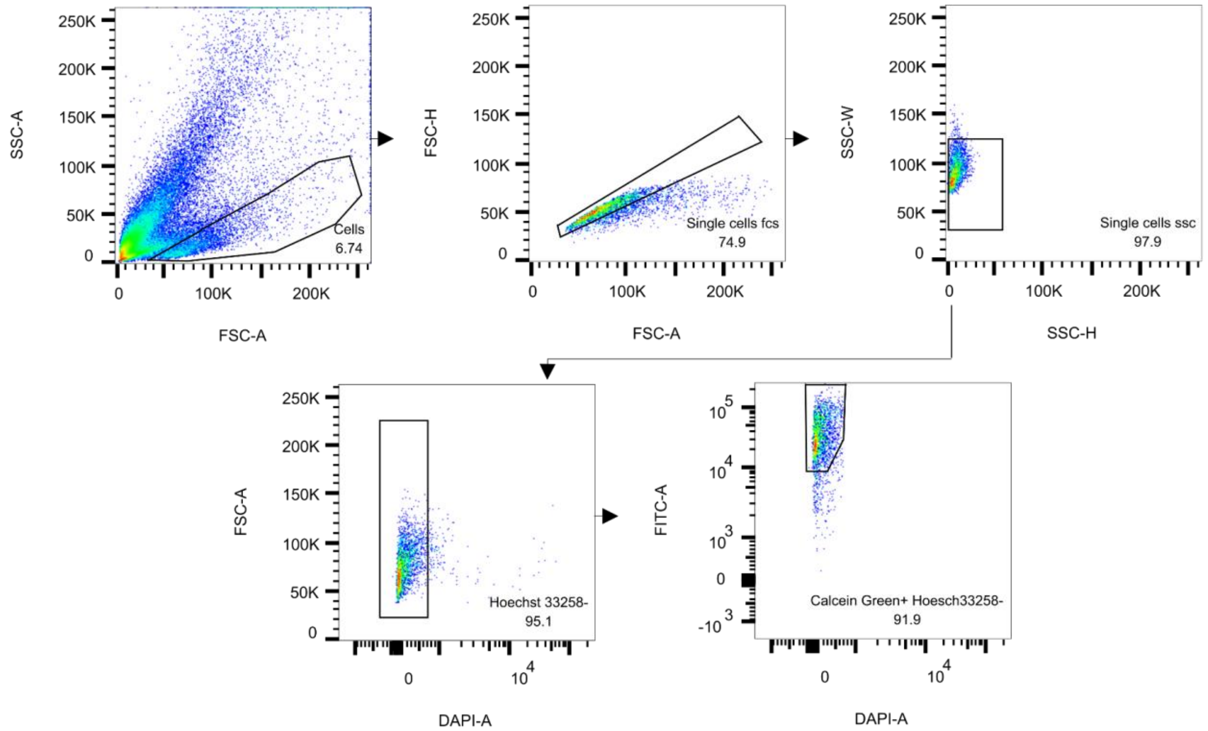

B

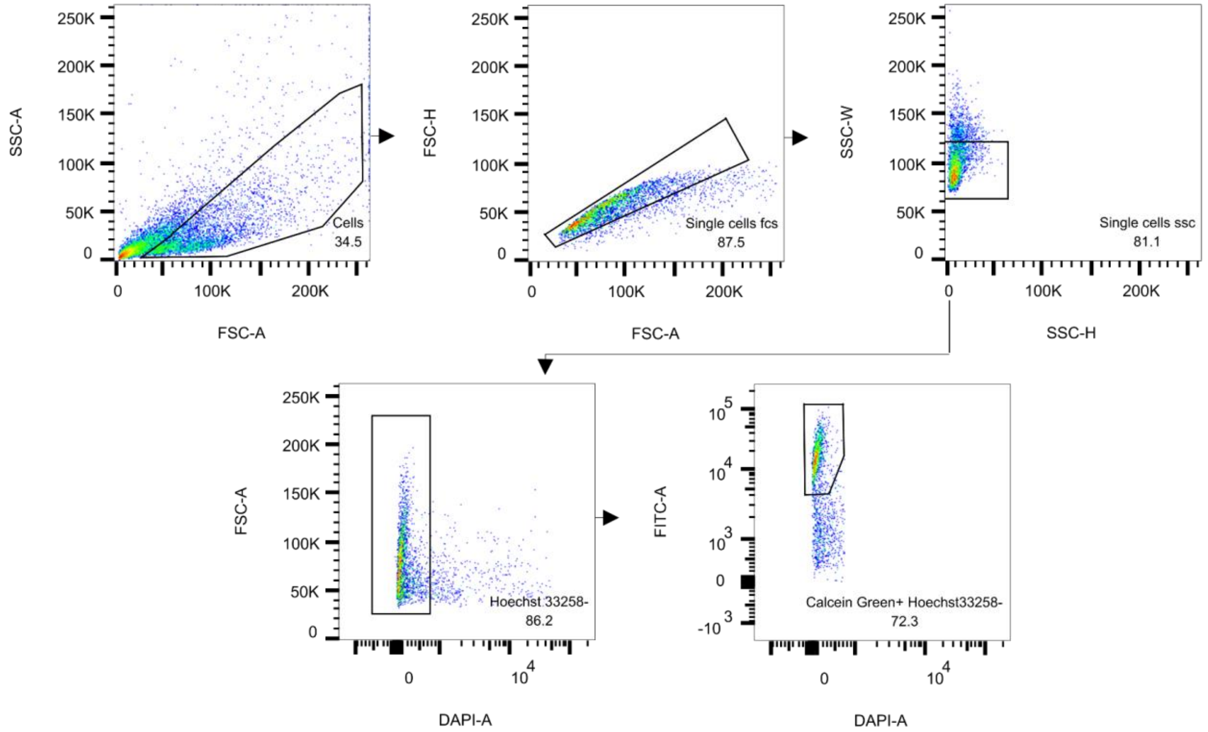

SFig. 3: Gating strategy for preparative single-cell sorting. Hierarchical gating strategy used for the isolation of viable single cells into 384-well plates for downstream sc-qPCR from **(A)** Miltenyi and **(B)** WorthGentle protocols. Suspensions were sequentially resolved through: (1) FSC-A vs. SSC-A for initial cell selection and debris exclusion; (2) FSC-A vs. FSC-H and (3) SSC-H vs. SSC-W for rigorous doublet exclusion; (4) DAPI-A vs. FSC-A to isolate intact, Hoechst 33258^{-} viable cells; and (5) FITC-A vs. DAPI-A to select Calcein Green^{+} Hoechst 33258^{-} live single cells for index sorting. Single-color controls and unstained cell suspensions were used to set baseline PMT detector voltages and establish background fluorescence thresholds for Hoechst 33258 and Calcein Green. Cells were sorted on a BD FACSMelody cell sorter and representative gating plots were rendered using FlowJo software (v10.9.0). *Abbreviations: DAPI-A – 4',6-diamidino-2-phenylindole area (capturing Hoechst 33258 fluorescence), FITC-A – fluorescein isothiocyanate area, FSC-A – forward scatter area, FSC-H – forward scatter height, SSC-A – side scatter area, SSC-H – side scatter height, SSC-W – side scatter width*.
